# Reply to MacColl: Repeated evolution from standing genetic variation

**DOI:** 10.1073/pnas.2605003123

**Published:** 2026-03-27

**Authors:** Marius Roesti, Jeffrey S. Groh, Felicity C. Jones, Catherine L. Peichel, Dolph Schluter

**Affiliations:** ^a^Division of Evolutionary Ecology, Institute of Ecology and Evolution, University of Bern, Bern 3012, Switzerland; ^b^Miller Institute for Basic Research in Science, UC Berkeley, CA 94720; ^c^Groningen Institute of Evolutionary Life Sciences, University of Groningen, Groningen 9747AG, Netherlands; ^d^Zoology Department and Biodiversity Research Centre, University of British Columbia, Vancouver, BC V6T 1Z4, Canada

MacColl (1) suggests that reproductive isolation and divergence in our stickleback study populations (2) predate postglacial lake colonization and were already present in two distinct colonizing lineages. Much evidence contradicts this view and instead supports repeated evolution in lakes that is largely driven by selection on old standing genetic variation brought by marine colonists.

## Repeated Evolution from Standing Genetic Variation

Repeated freshwater adaptation has been observed in real time, with evolution toward more benthic-like phenotypes and genotypes via standing genetic variation in mere decades (e.g., ref. 3). The same trends occur in ancient DNA samples of lake colonists at the end of the ice age (4). Modern marine and freshwater populations hybridize wherever they come into contact, and standing genetic variation for freshwater alleles is found in marine fish (e.g., ref. 3). Irrespective of possible population structure within the sea leading to variation in standing genetic variation, Pacific marine populations are phenotypically similar and reproductively compatible with each other and with the common Atlantic marine stickleback that split from the Pacific lineage longer ago (5).

## Continuous Phenotypic and Genomic Variation

Freshwater populations do not fall into two discrete clusters, and sculpin- and solitary-lake populations span a continuum of phenotypic and genomic divergence with varying reproductive isolation (2, 6). Our genomic phylogeny of these populations closely approximates a star, the model of phylogenetic independence and rapid divergence, rather than two discrete clusters (2). mtDNA also does not support the two lineage model (7). Thus, phenotypic and genetic data support rapid and repeated divergence of freshwater populations in postglacial lakes in response to sculpin-mediated selection (6, 8).

## Further Difficulties with MacColl’s View

MacColl presents no evidence for preexisting, reproductively isolated lineages within the sea, nor does he explain how two such lineages could both have rapidly colonized geographically interspersed and widespread postglacial lakes. Importantly, MacColl’s model also does not explain the remarkable coincidence required to yield a consistent association between stickleback phenotypes, reproductive isolation, and sculpin presence (2, 6, 7) without significant independent within-lake evolution. MacColl invokes the sympatric Limnetic–Benthic stickleback species pairs, which we did not examine in our study and are phenotypically distinct from sculpin-sympatric and solitary populations (8).

## MacColl’s Figure Is Misleading

**Fig. 1.**
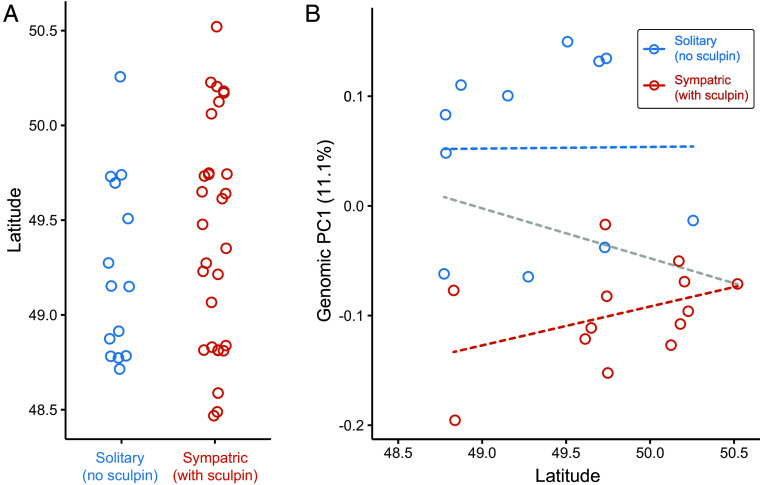
Latitude does not explain sculpin co-occurrence or associated stickleback divergence. (*A*) Across 43 published stickleback lakes with reported sculpin presence or absence (from refs. 2, 6, 7, 10, and 11), there is no association with latitude. (*B*) The major axis of genomic divergence among stickleback from sculpin-present and sculpin-absent lakes—calculated from autosome-wide SNPs across 24 populations (2)—varies continuously and is unrelated to latitude, both within lake types and across all lakes (dashed regression lines; all *P* > 0.17).

The y-axis of MacColl’s figure is not the probability of being “benthic-like” but simply contrasts populations from a subset of lakes with and without sculpin. He omits the broader distribution of lakes, which shows no relationship between latitude and sculpin presence (Fig. 1*A*) nor population genomic divergence (Fig. 1*B*). Notably, benthic-like freshwater populations occur far north of the proposed “southern lineage,” including in Alaska (9). Thus, divergence among freshwater phenotypes in the region does not correspond to a single axis and cannot be reduced to a simple north–south split.

We agree with MacColl that the origin of standing genetic variation in the sea is interesting and requires further study. Yet, understanding early speciation requires recognizing the evidence that adaptation is rapid and repeatable, resulting in a continuum of divergence rather than typological categories.
